# Identification and Characterization of Vancomycin-Resistant *Staphylococcus aureus* CC45/USA600, North Carolina, USA, 2021

**DOI:** 10.3201/eid3101.241573

**Published:** 2025-01

**Authors:** Jennifer K. MacFarquhar, Anumita Bajpai, Teresa Fisher, Chad Barr, Alyssa G. Kent, Susannah L. McKay, Davina Campbell, Amy S. Gargis, Rocio Balbuena, David Lonsway, Maria Karlsson, Maroya Spalding Walters, D. Cal Ham, William A. Glover

**Affiliations:** Author affiliations: Centers for Disease Control and Prevention, Atlanta, Georgia, USA (J.K. MacFarquhar, A.G. Kent, S.L. McKay, D. Campbell, A.S. Gargis, R. Balbuena, D. Lonsway, M. Karlsson, M.S. Walters, D.C. Ham); North Carolina Department of Health and Human Services, Raleigh, North Carolina, USA (A. Bajpai, T. Fisher, W.A. Glover); Caldwell County Health Department, Lenoir, North Carolina, USA (C. Barr)

**Keywords:** VRSA, bacteria, antimicrobial resistance, staphylococci, vancomycin, CC45/USA600, resistant Staphylococcus aureus, VanA, public health, North Carolina, United States

## Abstract

Vancomycin-resistant *Staphylococcus aureus* (VRSA) is a rare but serious public health concern. We describe a VRSA case in North Carolina, USA. The isolate from the case belonged to the USA600 lineage and clonal complex 45. No transmission was identified. Confirmed VRSA cases should include a thorough investigation and public health response.

On December 3, 2021, the Centers for Disease Control and Prevention (CDC) confirmed a vancomycin-resistant *Staphylococcus aureus* (VRSA) isolate from a resident of North Carolina, USA. That isolate represented the 16th confirmed VRSA case identified in the United States (*1*,*2*). Although no transmission was identified in previous cases, CDC recommends a public health response to each confirmed case because of the potential for transmission and the serious clinical implications of widespread vancomycin resistance in *S. aureus* (*3*).

The patient was a 55-year-old man with a history of diabetes mellitus, hypertension, arthritis, pulmonary disease, peripheral vascular disease, methicillin-resistant *S. aureus* (MRSA), and vancomycin-resistant enterococci (VRE). The patient resided in a skilled nursing facility (SNF) for the 28 days before the incident specimen was collected. In the 60 days before specimen collection, the patient had acute care hospital (ACH) and SNF admissions, received care for a nonhealing foot wound at a wound care clinic (WCC), and received 5 antimicrobial agents, including vancomycin. The patient was in a private room and on contact precautions during all facility admissions for the 12 months before the positive VRSA identification. Cultures from the patient’s nonhealing foot wound, which was suspected of being infected, yielded the incident specimen.

The suspect isolate underwent species confirmation, vancomycin resistance screening, and antimicrobial susceptibility testing (*4*) by the North Carolina State Laboratory for Public Health (Appendix). CDC performed short-read whole-genome sequencing and genome assembly, staphylococcal cassette chromosome *mec* and protein A (*spa*) typing, multilocus sequence typing, and whole-genome multilocus sequence typing.

The confirmed VRSA isolate demonstrated resistance to vancomycin (MIC 64 µg/mL by gradient diffusion, 128 µg/mL by broth microdilution) (*4*). Whole-genome sequencing analysis identified the presence of *mecA* and *vanA* genes. The *vanA* gene is likely plasmid-encoded on the basis of the similarity of its genomic context to other plasmid-encoded *vanA* genes in publicly available data. Typing results indicated the isolate was *spa* type t1081, staphylococcal cassette chromosome *mec* type V, and sequence type 45, belonging to the USA600 lineage and clonal complex 45 (CC45/USA600) (*5*) (Figure).

**Figure:**
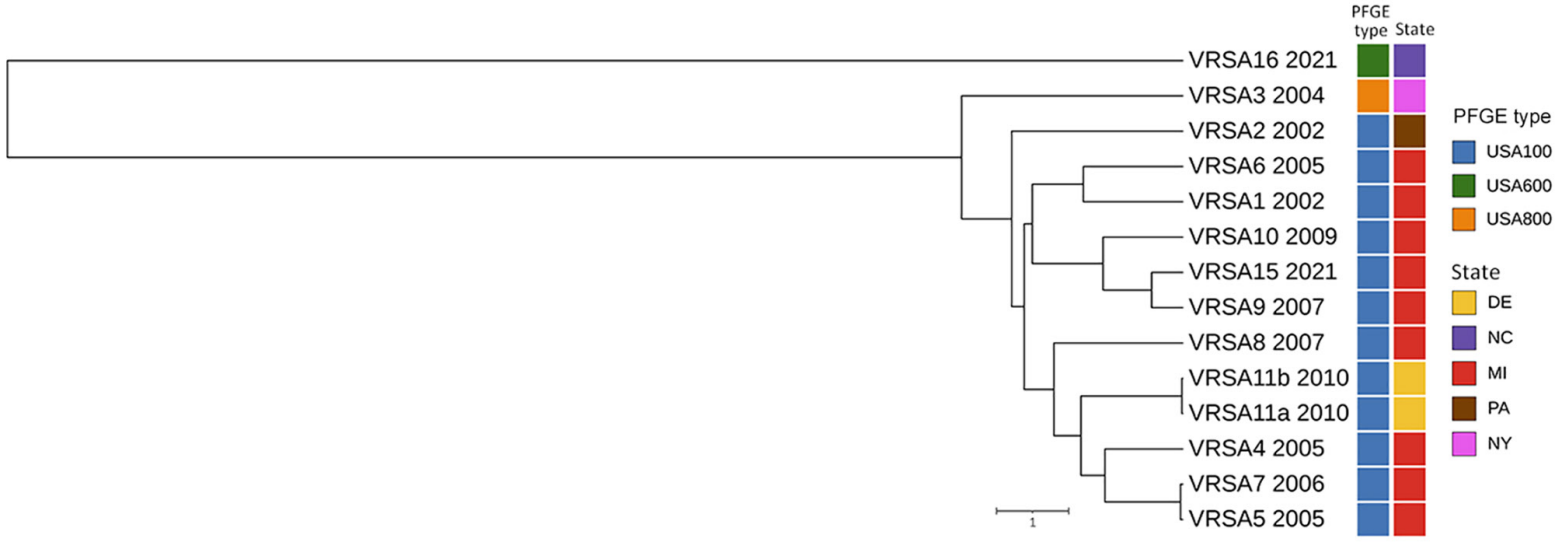
Whole-genome multilocus sequence typing for identification and characterization of VRSA, North Carolina, USA, 2021. Unweighted pair group method with arithmetic mean dendrogram shows the relationship of VRSA16 and previously sequenced VRSA genomes from US patients; sequence data for VRSA 12 (CC5/PFGE type unknown), VRSA 13 (CC30/USA1100), and VRSA 14 (CC5/USA100) were not available. Date of isolation (year), PFGE type (also known as USA type), and geographic location of each VRSA isolate are indicated. Scale bar indicates the whole-genome multilocus sequence typing allelic distance. PFGE, pulsed-field gel electrophoresis; VRSA, vancomycin-resistant *Staphylococcus aureus*.

We conducted site visits to the ACH, WCC, and SNF that provided care to the patient during the 60 days before collection of the positive specimen. We identified minimal infection prevention and control gaps at the ACH and WCC; at the SNF, we observed inappropriate use or absence of personal protection equipment, low adherence to hand hygiene, poor wound care technique, inability to outline cleaning and disinfection protocols, and crowded/cramped spaces with minimal access to hand hygiene stations (e.g., lack of handwashing sinks and alcohol-based hand sanitizers). The SNF had no dedicated infection preventionist.

We defined contacts as persons having extensive or moderate interaction (*3*) with the patient or the patient’s environment during the 60 days before the specimen collection date. We collected screening specimens from the nares, axilla, groin, and wounds (if present) of contacts using 1 ESwab (COPAN, https://www.copanusa.com) per site, with the exception of the axilla and groin, which could be combined. We identified 115 contacts: 83 staff from the ACH and WCC, 12 SNF staff, 16 SNF residents, and 4 patient household members. No ACH or WCC patient contacts were identified. We collected 228 specimens from 110 contacts: 83 ACH and WCC staff, 23 from the SNF (9 staff and 14 residents), and 4 household members. Among 224 screening specimens (109 nares, 109 axilla/groin, and 6 wound) that met acceptance criteria from 109 contacts, no VRSA was isolated. After 49 days in the ACH and beginning 1 week after completion of treatment for VRSA with meropenem and daptomycin, the patient had negative serial cultures over the next 3 weeks collected from the nares, axilla, groin, and wound and was discharged back to the SNF.

Since VRSA was identified in the United States in 2002, confirmed cases are uncommon. The case reported here is notable for its location in the southern United States and belonging to the globally distributed CC45. In contrast to prior VRSA cases (*1*,*6*) with strains primarily associated with healthcare (*5*), CC45 circulates in both healthcare facilities and community settings (*7*). Similar to prior cases (*2*), this patient had multiple underlying conditions and a history of MRSA and VRE, supporting the hypothesis that VRSA resulted from conjugal transfer of the *vanA* gene from VRE to MRSA (*8*).

As for other VRSA investigations (*6*), we did not identify transmission, which is notable here given the identified infection prevention and control gaps. One possible explanation for the lack of transmission is that MRSA isolates harboring the *vanA* gene (VRSA) may be less fit or less transmissible. At least 1 laboratory study showed reduced fitness of VRSA isolates after vancomycin exposure (*9*), which might have contributed to the lack of transmission here.

In conclusion, emergence of this unique VRSA strain highlights the potential for emergence of other novel transmissible strains. Although the lack of transmission is reassuring, continued vigilance and investigation for all confirmed cases is paramount given the potential for vancomycin resistance to emerge in different *S. aureus* lineages, thereby resulting in novel strains that are more fit and thus more transmissible.

Appendix Additional information on identification and characterization of vancomycin-resistant *Staphylococcus aureus* CC45/USA600, North Carolina, USA, 2021.
