# Supplementary material for: Identification and Characterization of Vancomycin-Resistant Staphylococcus aureus CC45/USA600, North Carolina, USA, 2021
Source: Emerg Infect Dis. 2025 Jan;31(1):194–6. doi: 10.3201/eid3101.241573 (PMC11682812; doi:10.3201/eid3101.241573)
Supplement: Appendix — Additional information on identification and characterization of vancomycin-resistant Staphylococcus aureus CC45/USA600, North Carolina, USA, 2021. [file 24-1573-Techapp-s1.pdf]

Article DOI: https://doi.org/10.3201/eid3101.241573

EID cannot ensure accessibility for supplementary materials supplied by authors. Readers who have difficulty accessing supplementary content should contact the authors for assistance.

Identification and Characterization of Vancomycin-Resistant *Staphylococcus aureus* CC45/USA600, North Carolina, USA, 2021

Appendix.

Supplementary Laboratory Methods for Specimen Collection, Isolate Identification, Antimicrobial Susceptibility Testing, and Whole-Genome Sequencing and Analyses

An ESwab 493C02 (COPAN) containing liquid Amies medium was used to collect screening specimens. Vancomycin susceptibility testing was conducted using a gradient diffusion strip (Etest; bioMérieux) and the Sensititer Gram Positive FDANDPF (Thermo Fisher Scientific) broth microdilution (BMD) panel according to the manufacturers' specifications. For confirmatory testing, North Carolina State Laboratory for Public Health sent the isolate to the CDC for organism identification by using matrix-assisted laser desorption ionization–time-of-flight mass spectrometry and reference antimicrobial susceptibility testing by using CDC's in-house–developed BMD panel based on Clinical and Laboratory Standards Institute (CLSI)-established criteria (*1*). Short-read whole-genome sequencing was performed by using an Illumina Miseq System. Genomic DNA was extracted from colonies cultured overnight on sheep-blood agar (SBA) by using the Promega Maxwell 16 Cell Low Elution volume DNA Purification Kit and Maxwell 16 MDx Instrument. Genomic DNA was sheared to a mean size of 600 bp by using a Covaris LE220 focused ultrasonicator. DNA fragments were cleaned with Mag-bind beads (Omega Bio-tek), dual-indexed sequencing libraries were prepared by using NEBNext Ultra library prep reagents (New England Biolabs), and barcoding indices were synthesized in the CDC Biotechnology Core Facility. Libraries were analyzed for size and

concentration, normalized, pooled, and denatured for loading onto flowcells for cluster generation. Sequencing was performed on a Miseq by using Miseq 2 × 250-bp paired-end sequencing kits (Illumina). On completion, sequence reads were filtered for read quality, basecalled, and demultiplexed by using bcl2fastq (v2.20). Assembly and multilocus sequence typing (MLST) were performed by using CDC's QuAISAR-H pipeline (https://github.com/DHQP/QuAISAR_singularity). Whole-genome multilocus sequence typing (wgMLST) was performed by using BioNumerics (v7.6) and visualized as an unweighted pair group method with arithmetic mean dendrogram by using interactive tree of life (iTOL, v5). Previously available sequenced VRSA genomes (*2*) were included in the wgMLST analysis; sequence data for VRSA 12 (CC5/pulsed-field gel electrophoresis [PFGE] type unknown), VRSA 13 (CC30/USA1100), and VRSA 14 (CC5/USA100) were not available (*3*). Isolates with previously unknown PFGE types were assigned inferred PFGE types (https://www.cdc.gov/hai/settings/lab/ccalgorithm.html) on the basis of sequence type, clonal complex, staphylococcal cassette chromosome *mec* type, and wgMLST results. Sequences were deposited in the NCBI Sequence Read Archive under BioProject no. PRJNA533550 with BioSample accession no. SAMN44571444.

To suppress normal flora while simultaneously enriching for possible VRSA, 3 mL tryptic soy broth with 6.5% sodium chloride was inoculated with 100 µL of the patient's sample and incubated overnight (18–24 hours at 35°C ± 2°C ambient air). After incubation using a 10-µL loop, broth was plated on 3 different in-house prepared media: SBA, brain-heart infusion (BHI)-vanc (6 µg/mL) agar, and Columbia nalidixic acid (CNA). BHI-vanc plates were incubated at 35°C ± 2°C in ambient air, whereas the CNA and SBA plates were incubated at 35°C ± 2°C in CO_2 for up to 48 hours and observed for β-hemolysis and growth at both 24 and 48 hours.

Supplementary Laboratory Results

The isolate showed resistance to cefoxitin, penicillin, levofloxacin, tetracycline, and vancomycin (MIC of 128 µg/mL) and susceptibility to ceftaroline, chloramphenicol, clindamycin, daptomycin, doxycycline, erythromycin, gentamicin, linezolid, rifampin, tigecycline, and trimethoprim/sulfamethoxazole according to CLSI-established criteria (*1*).

Supplementary Infection Prevention and Control Recommendations

1) Reeducate staff on appropriate wound care and infection prevention policies and practices, specifically hand hygiene and appropriate use/wear of surgical masks.
2) Provide routine scheduled continuing education and just-in-time reeducation for all staff on infection control policies and practices and provide regular reinforcement.
3) Conduct routine hand hygiene, personal protection equipment, and wound care audits and provide feedback.
4) Install additional hand hygiene stations for staff use in resident areas.
5) Consult local health department to determine fire code and maximize opportunities for hand hygiene stations.
6) Install splash barriers at hand hygiene sinks where splashing could occur.
7) Review infection control policies and update as needed by using nationally recognized standards.
8) Ensure visiting dental hygienists follow appropriate CDC infection prevention practices for dental settings.

References

1. Clinical and Laboratory Standards Institute. Performance standards for antimicrobial susceptibility testing, 33rd edition (M100-S33). Wayne (PA): The Institute; 2021.

2. Kos VN, Desjardins CA, Griggs A, Cerqueira G, Van Tonder A, Holden MT, et al. Comparative genomics of vancomycin-resistant *Staphylococcus aureus* strains and their positions within the clade most commonly associated with methicillin-resistant *S. aureus* hospital-acquired infection in the United States. MBio. 2012;3:e00112–12. PubMed https://doi.org/10.1128/mBio.00112-12

3. Cong Y, Yang S, Rao X. Vancomycin resistant *Staphylococcus aureus* infections: A review of case updating and clinical features. J Adv Res. 2019;21:169–76. PubMed https://doi.org/10.1016/j.jare.2019.10.005
